# Physical Activity Recognition Based on a Parallel Approach for an Ensemble of Machine Learning and Deep Learning Classifiers

**DOI:** 10.3390/s21144713

**Published:** 2021-07-09

**Authors:** Mariem Abid, Amal Khabou, Youssef Ouakrim, Hugo Watel, Safouene Chemcki, Amar Mitiche, Amel Benazza-Benyahia, Neila Mezghani

**Affiliations:** 1 Laboratoire LIO, Centre de Recherche du CHUM, Montreal, QC H2X 0A9, Canada; oua.youssef@gmail.com (Y.O.); watelhugo@gmail.com (H.W.); neila.mezghani@teluq.ca (N.M.); 2 LICEF Institute, TELUQ University, Montreal, QC G1K 9H6, Canada; safouene.chemkhi@supcom.tn; 3 INRS, Centre Énergie, Matériaux et Télécommunications, Montreal, QC G1K 9A9, Canada; mitiche@emt.inrs.ca; 4 Département de Génie des Systémes, École de Technologie Supérieure (ÉTS), Montreal, QC H3C 1K3, Canada; 5 LR11TIC01, COSIM Lab., University of Carthage SUP’COM, El Ghazala 2083, Tunisia; benazza.amel@supcom.rnu.tn

**Keywords:** machine learning, deep learning, big data, data streams, Internet of things, sensor data, intelligent systems, multivariate time series, tensor

## Abstract

Human activity recognition (HAR) by wearable sensor devices embedded in the Internet of things (IOT) can play a significant role in remote health monitoring and emergency notification to provide healthcare of higher standards. The purpose of this study is to investigate a human activity recognition method of accrued decision accuracy and speed of execution to be applicable in healthcare. This method classifies wearable sensor acceleration time series data of human movement using an efficient classifier combination of feature engineering-based and feature learning-based data representation. Leave-one-subject-out cross-validation of the method with data acquired from 44 subjects wearing a single waist-worn accelerometer on a smart textile, and engaged in a variety of 10 activities, yielded an average recognition rate of 90%, performing significantly better than individual classifiers. The method easily accommodates functional and computational parallelization to bring execution time significantly down.

## 1. Introduction

Miniaturization of complex electrical devices at continually lower cost has brought about the development of a variety of wearable sensors and their embedding in healthcare-dedicated Internet of things (IoT). The broad purpose of a healthcare IoT, sometimes called Internet of medical things, abbreviated as IoMT, is to provide a network of embedded systems to acquire, communicate, and analyze data for remote medical practice of accrued quality. Sensors in the embedded systems of IoMT can perform a variety of useful measurements, such as heart rate, body temperature, blood pressure, temporal data, as with electrocardiography (ECG), and activity data, such as movement acceleration. The purpose of this study is to investigate a human activity recognition (HAR) method of accrued decision accuracy and speed of execution to be applicable and practicable in healthcare IoT applications. The method uses acceleration data of human movement recorded by a single, comfortably worn accelerometer. Recent HAR research in healthcare has used various wearable sensors for health monitoring and physical rehabilitation systems [1]. For instance, ref. [2] provided a qualitative synthesis of studies using wearable body sensors for health monitoring, pointing out a number of shortcomings in prior research with respect to both sample size and participant demographics. Such systems aim at developing methods for automatically recognizing human physical activities by analyzing data gathered by sensors in wearable devices. The basic problem is to assign a time series segment of sensor data to a corresponding activity during that time segment [3].

Machine learning can model a wide range of human physical activities for HAR in wearable-sensor embedded systems. However, serious challenges remain. First, both training and learning technique evaluation require large annotated data sets. This can be both a data-intensive and computation-intensive process. Thus, it is important to design parallel algorithms that fully exploit the computational capacity of the target machine and reduce the training time. Moreover, technical issues such as parallel ensemble learning algorithms that aim at optimizing both the accuracy and computational costs are not yet fully addressed by previous research works.

There have been several HAR studies using wearable sensors that we detail in the next section. The results of current research firmly establish the merit and feasibility of wearable sensor HAR and justify further investigation to develop practicable, parallel efficient algorithms. The purpose of this study is to investigate a parallel classifier combination method toward this end. Specifically, the contributions of this study are:

- A large dataset to serve HAR system development, recorded on participants using a comfortable smart textile garment with an embedded single waist-worn accelerometer.
- A parallel architecture to combine traditional and deep learning pattern classification algorithms, for accrued computational and classification accuracy, that we referred to as an ensemble learning architecture. This architecture includes both the training and testing aspects of algorithm development, for ease of application development.
- A parallel implementation of this ensemble learning architecture.

The proposed ensemble architecture is novel because it combines feature engineering and feature learning, and does so with a parallel implementation of cross-validation: it fuses the decisions of distinct classifiers in a manner that increases the robustness and accuracy of the final decision. The method is demonstrated in the important field of health monitoring and rehabilitation. It is tractable so as to easily allow the inclusion of additional classification models. The remainder of this paper is organized as follows: Section 2 gives an overview of sensor-based HAR systems, Section 3 presents a detailed description of the materials and methods used in this study, which includes data acquisition, preprocessing, and the proposed parallel architecture framework. Section 4 describes and discusses the experimental results. Finally, Section 5 contains a conclusion and perspectives for further research.

## 2. Related Work

This paper deals with the problem of wearable sensor-based HAR to recognize activity data collected from a single accelerometer embedded in a wearable device using an ensemble architecture that combines both feature engineering and feature learning approaches with a parallel implementation of the leave-one-subject-out cross-validation method. We highlight in this section the particularities of this work, in terms of research goals and contributions, compared to the related work of wearable sensor-based HAR.

Sensor-based HAR can be defined as the process of interpreting sensor data to recognize a set of human physical activities [4]. More specifically, sensor-based HAR is a classical multivariate time series analysis problem that aims at classifying contiguous portions of sensor data streams which cover activities of interest for a given target application.

Several approaches have been proposed for wearable sensor-based HAR, in which various human activities were investigated, including common daily activities, ambulation, and fitness exercise, and they have been well surveyed. Lara et al. [5] surveyed the state of the art in HAR based on wearable sensors. The authors evaluated the state-of-the-art HAR systems by defining a taxonomy that allows one to compare and analyze them within groups that share common characteristics such as the recognized activities, the type of sensors and the measured attributes, the integration device, the obtrusiveness level, the type of data collection protocol, the energy consumption level, the classifier flexibility level, the feature extraction method, the learning algorithm, and the overall accuracy for all activities. Twenty eight HAR systems present in the literature have been compared according to the aforementioned aspects. Attal et al. [6] presented a review of recognition of human activities using wearable sensors and focused on wearable sensors’ placement, preprocessing data including feature extraction and selection and classification techniques. Nweke et al. [7] reviewed deep learning methods for mobile and wearable sensor-based human activity recognition. The review presents the methods and their advantages and limitations. Wang et al. [8] presented a survey on the wearable sensor modality centred HAR in health care, including the sensors used in HAR, the sensor placement on different body parts, the most commonly seen sensor platforms in HAR, activities defined in this field, data segmentation, feature learning, classification, etc.

As expected, most of the sensor measurements used in the existing studies are categorized as related to the user’s movement (e.g., using accelerometers or GPS), environmental variables (e.g., temperature and humidity), or physiological signals (e.g., heart rate or electrocardiogram). These are typically collected from a small number of subjects and in restrictive settings. For instance, Bao and Intille [9] reported the number of subjects who participated in past studies on activity recognition using acceleration as ranging from 1 to 24. In a more recent review, Wang et al. [10] presented several widely used public HAR datasets of acceleration samples and reported the number of subjects who participated in these studies, ranging from 1 to 36. Moreover, these approaches vary depending on the sensor technologies used to acquire the data, the features used to train the model: handcrafted feature extraction (feature engineering) or automatic feature extraction (feature learning), and the learning-based classification techniques, namely classical machine learning and deep approaches.

The majority of the research works on wearable sensor-based HAR have so far focused on using accelerometers. They are particularly effective in monitoring activities that involve repetitive body motions, such as walking, running, sitting, standing, and climbing stairs.

Smartphones and wearables equipped with accelerometers, such as smart clothes, are widely used modalities in sensor-based HAR. Garcia et al. [11] discussed publicly available datasets collected from different wearables and/or smartphone sensors such as the WISDM [12], PAMAP2 [13], and MHealth [14] datasets. Particularly, for the smartphone-based HAR, a review of related work was presented in [15], with an emphasis on the activities analysed, types of sensor data used, features extracted, classification method applied, and accuracy achieved. For instance, Kwapisz et al. [12] collected sensor data from phone-based accelerometers using an application installed on each user‘s phone. Three-axis accelerometer sensors were collected from 36 users carrying a smartphone placed on their front pants pocket. Bayat et al. [16] collected three-axis accelerometer sensor data from 4 subjects, each carrying a cell phone, in either the in-hand or in-pocket position. Nowadays, almost everyone carries a smartphone, which provides a convenient way to collect raw sensor data to infer physical activity. However, one drawback is that smartphones are usually carried in bags or pockets, and generally not positioned on the body.

Another approach to detect a broad range of physical activities is to wear multiple accelerometers placed simultaneously at different locations on the subject body (hip, chest, ankle, waist, etc.). For instance, Kern et al. [17] placed three-axis accelerometer sensors in all major joints on the human body to recognize everyday postures and activities: just above the ankle, just above the knee, on the hip, on the wrist, just above the elbow, and on the shoulder. Bao and Intille [9] collected sensor data from five biaxial accelerometers placed on each subject’s right hip, dominant wrist, non-dominant upper arm, dominant ankle, and non-dominant thigh to recognize ambulation, posture, and other everyday activities. Even though this approach is known for yielding strong performance rates, it is not practical in clinical settings, for instance.

Other studies, like ours, have focused on the use of a single waist-worn accelerometer for the activity recognition. For example, Bidargaddi et al. [18] analyzed acceleration signals recorded with a three-axis accelerometer, which is mounted on the waist of patients undergoing cardiac rehabilitation.

The ideal sensor location for particular applications is still a subject of much debate. Bao and Intille [9] concluded that the accelerometer placed on the thigh was the most powerful for distinguishing between a range of common everyday household activities. Cheung et al. [19] concluded that the use of a single waist-mounted triaxial accelerometer is the most practical solution for health monitoring. Cleland et al. [20] concluded that the sensor placed on the hip provides the best measures to recognize most everyday activities.

In general, a HAR system is composed of the following steps: (1) sensor data time series acquisition, (2) raw data preprocessing, (3) time series segmentation, (4) feature engineering or learning and feature selection, and (5) classification.

First, wearable sensors collect time series data from users. Then, the raw data collected is processed and represented in the form of labeled multivariate time series.

The purpose of data fusion is to combine data acquired by different sensors or in different axes to increase the reliability, robustness and generalization ability of HAR systems. Data fusion can be achieved at the following three levels: data level, feature level, and decision level [21]. Some existing methods allowing one to fuse different data modalities for human activity recognition include the weighted average and least square method, Kalman filtering and Dempster-Shafer theory [22]. Another common method is to consider the data acquired by different sensors or in different axes, i.e., the multivariate time series, individually in a separate channel, and consider each channel separately as an input of the models [23]. Cai et al. [24] considers three-axis data of a tri-axial accelerometer as an integration and classifies activities based on the resultant acceleration. In the feature-level fusion, conventional feature fusion methods simply concatenate several kinds of extracted features together. For instance, Fu et al. [25] proposed to fuse multiple features in a generalized subspace learning framework. Tao et al. [26] selected the representative frequency features of acceleration along each axis, respectively, and combined them into a vector. The decision-level fusion involves systematic fusion of individual classifier decisions to obtain a final decision in order to increase the accuracy, robustness and generalization [27]. The commonly used classifier combination schemes are simple voting, majority voting, weighted majority, fusion score and posterior probability [28].

Segmentation is the process of dividing the continuous data stream into smaller data segments. Most of the segmentation techniques can be divided into three categories: activity-defined windowing, event-defined windowing, and sliding windowing. The sliding window strategy, which allows a degree of overlap between fixed-size windows, has been prevalent [14].

Feature engineering uses domain knowledge to define the features of data representation [29], and feature learning determines a mapping from the data domain to the feature domain without relying on any domain knowledge. Current research has not clearly established which of feature engineering or feature learning is more potent in HAR. Figo et al. [30] discussed several feature engineering schemes for accelerometer data in HAR, including time domain, frequency domain and discrete domain metrics. Plötz et al. [3] evaluated the effectiveness of feature learning for physical activity classification tasks without involving domain-specific knowledge, compared to feature engineering techniques. He et al. [31] proposed a HAR system based on discrete cosine transform (DCT) as an automatic feature extraction technique. Atallah et al. [32] investigated three methods of feature selection, namely Relief, Simba, and Minimum Redundancy Maximum Relevance (MRMR) methods.

The issue of recognizing physical activities on the basis of accelerometer data has been solved many times by different classification approaches, e.g., k-Nearest Neighbour (k-NN) [33], Decision Trees (DTs) [34], Support Vector Machines (SVMs) [35], Artificial Neural Networks (ANNs), deep learning [3], the hidden Markov model [36], as well as ensemble learning techniques, such as in [37]. Bayat et al. [16] combined three classifiers by the average of the probability fusion method, using accelerometer data from smartphones on two datasets: in-hand phone position and in-pocket phone position. For in-hand phone position, the classifiers consist of the multilayer perceptron, LogitBoost and SVM classifiers, with 91.15% accuracy. For the in-pocket phone position, the best combination is of multilayer perceptron, random forest and simple logistic with 90.34% accuracy. Catal et al. [38] combined three classifiers by the average of probabilities fusion method using accelerometer data from smartphones (the WISDM dataset [12]). The classifiers consist of the J48, logistic regression, and MLP classifiers. Experimental results showed that this new model exhibited a better performance than the standalone classifiers.

This study differs from most prior work in that we use a single device conveniently worn rather than multiple devices distributed across the body, and we require neither additional actions by the user nor any additional specialized equipment. Moreover, this approach enables practical real-world applications for the proposed models, specifically in health monitoring and rehabilitation. Furthermore, we have generated and tested the proposed models using more users than most of the previous studies in accelerometer sensor-based HAR we reviewed and expect this number to grow substantially since we are continuing to collect data. In the light of the previous investigated studies, we proposed ensemble architecture that combines feature engineering and feature learning techniques, which not only allows us to compare heterogeneous classifiers, but also combines their predictions. Obviously, the ensemble learning architectures for human activity recognition have been used many times before and it can be concluded from the literature that ensemble methods produce better results compared to standalone algorithms [37]. However, what is new in this approach is that first, we ran a large database using a parallel implementation of cross-validation to estimate the generalization capabilities of the proposed learning techniques, and second, we applied different fusion approaches (data, feature and decision) all along the pipelines.

## 3. Materials and Methods

**Figure 1:**
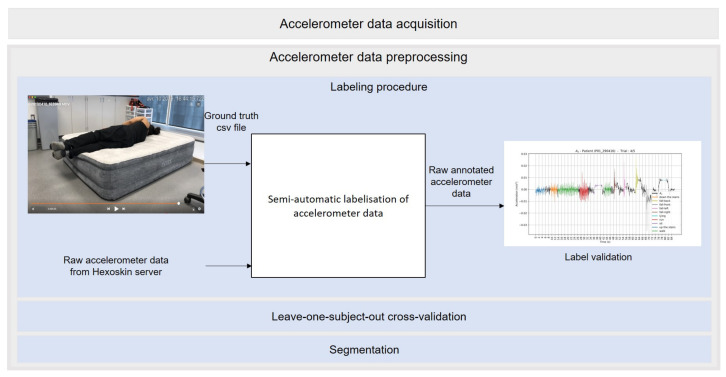
The detailed preprocessing steps of raw accelerometer data.

Figure 1 shows the different steps of the overall process, from data acquisition to segmentation and data labeling.

### 3.1. Data Acquisition

#### 3.1.1. Participants

Data acquisition is performed in two stages. At the first stage, a convenience sample of 14 healthy and young volunteers (age 25.43±7.51 years, weight 60.7±6.7 kg, and height 172.7±7.2 cm) is retained to participate in the data acquisition. At the second stage, 30 healthy and young volunteers (age 24.26±3.35 years, weight 68.4±12.3 kg, and height 170.3±0.08 cm) participate in the data acquisition. The two merged datasets will be the input of the HAR system for healthcare. The study is approved by the Ethics committee (ethical approval code: RDCPJ 522843-17) of the Centre Hospitalier de l’Université de Montréal (CHUM), the École de Technologie Supérieure (ÉTS), and the TÉLUQ university, Canada. Data collection was performed in the biomechanics laboratory on the 7th floor of the research center of CHUM (CRCHUM). Informed consent was obtained from all subjects involved in the study.

#### 3.1.2. Equipment

Data were acquired with a single waist-mounted three-axial accelerometer (a non-invasive sensor with a 13-bit resolution and a frequency of 64 Hz) embedded in a health monitoring wearable shirt, Hexoskin (Carré Technologies, Inc. in Montréal, QC, Canada). The latter also includes three other sensors to record cardiac and respiratory data. These sensor data are not used in the present study. The sleeveless shirt is made of a stretchable fabric (73% micro polyamide and 27% elastane), with anti-bacterial, breathable, lightweight, UV-protective and quick-dry properties. Thus, it is easy to put on, comfortable and can be used in any ambient environment. The accelerometer data acquisition can be performed continuously without hampering the movements of the person wearing it. When in use, the recording device connector slot is plugged into the shirt connector. Once connected, the accelerometer data are transmitted from the recording device to Hexoskin servers via Bluetooth and a dedicated cell phone application (the Hexoskin app), which outputs a report capturing health-related information of the performer. The data can be downloaded later to a PC via a USB cable and the HxServices software. The validity of the Hexoskin as an accelerometer-based physical activity monitor has been already approved [39].

#### 3.1.3. Research Protocol

**Figure 2:**
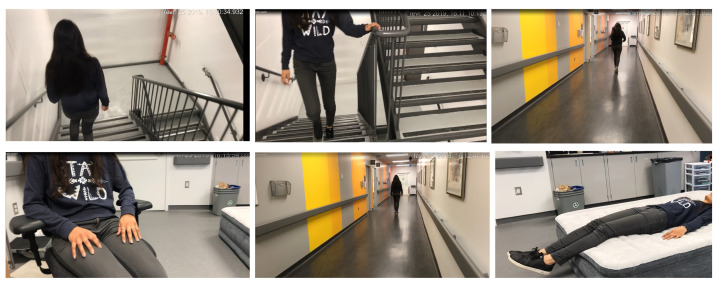
Experimental setup showing some of the performed physical activities in our research protocol: going down stairs, going up stairs, running, sitting, walking, and lying down.

The dataset used in this study is acquired by our research team. It will serve as a basis for the development of human physical activity recognition systems for medical purposes. In the experiments, 44 participants wearing the Hexoskin shirt, performed 10 activities. Before the acquisition, the participant anthropometrics were recorded (i.e., age, gender, height, weight). In order to capture the intra-subject variability and collect a big dataset, each subject was asked to perform 6 trials for each sequence of activities—that is, the participant performed the different activities in a different order in each trial. A sequence of the activities performed in the laboratory environment is: go up the stairs two floors (A1), go down the stairs two floors (A2), walk for 30 s along the corridor (A3), run for 30 s along the same corridor (A4), sit for 30 s on a chair (A5), fall-right, -left, -front and -black (respectively, A6 to A9), and lie intentionally on a mattress (A10) (Figure 2). The 11th class corresponds to transitional activities. This class was removed from the dataset before training.

We note that trials were video recorded using a separate smartphone. Then, the video was used as a benchmark to annotate the activities. Each video was tagged by a timestamp in milliseconds.

### 3.2. Preprocessing

#### 3.2.1. Labeling Procedure

**Figure 3:**
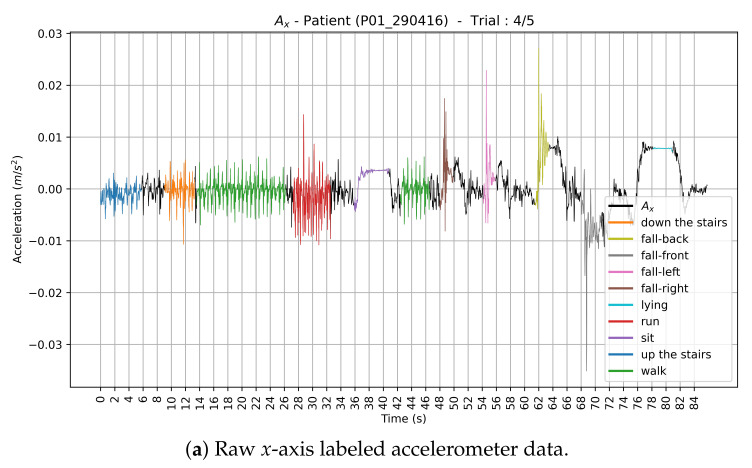

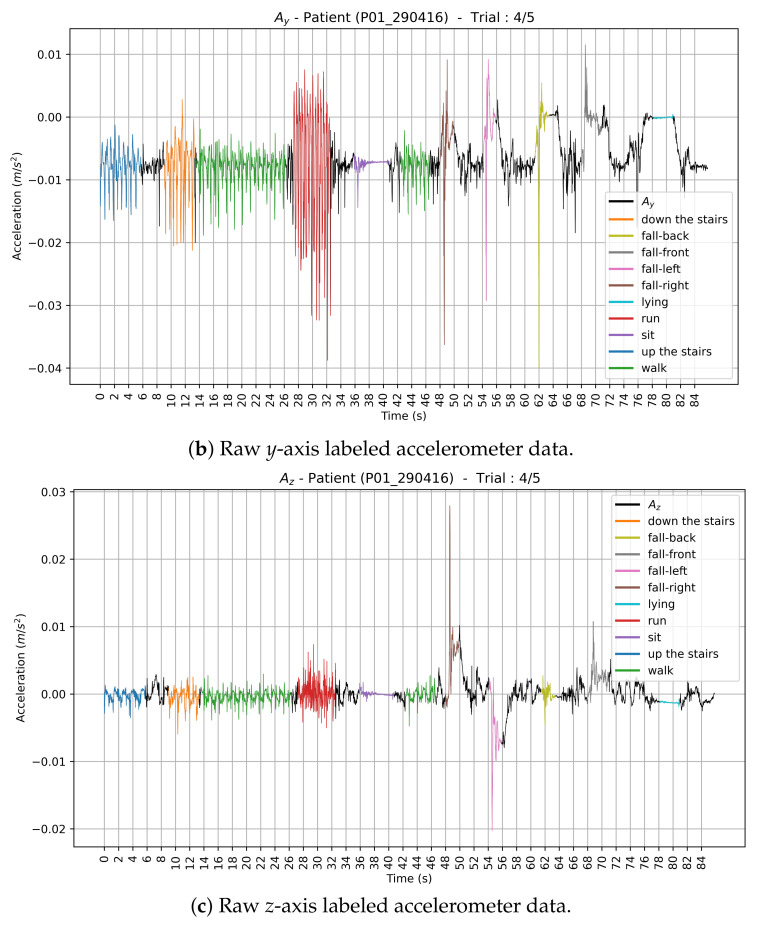
Plots of the raw three-axis labeled accelerometer data corresponding to one trial of a given participant (P01_290416). Each data cluster corresponding to an activity label has a different color on the plot.

First, two members of our research team analyzed each video. They manually and precisely annotated the temporal bounds of the observed activities (i.e., start and end times of each activity) in the video. Hence, a ground truth csv file was created for each video with the start and end times of the activities, as well as the class labels. Then, two other members of our research team verified the annotation process. The raw unlabeled accelerometer records of each axis were downloaded separately from the Hexoskin server. The first column represents the timestamp (in seconds) and the second column represents the data themselves. We retrieve the start date and time of the data acquisition from the “statistics.csv” file downloaded from the Hexoskin server. A second csv file was then automatically generated. In this file, the first column represents the timestamp (in milliseconds). Data from the *x*, *y*, and *z* axis were, respectively, in the 2nd, 3rd and 4th columns. The Euclidean norm was automatically computed and stored in the 5th column. Then, we automatically assigned a ground truth label to each timestamp of the data stream based on the ground truth csv file. To fine-tune the labeling procedure, we visually inspected the plots of the raw three-axis labeled accelerometer data. Each data cluster corresponding to an activity label has a different color on the plot, as depicted in Figure 3.

The raw data were processed using Matlab R2019a. All the raw and processed data were anonymized and stored in the server of the Imaging and Orthopaedics Research Laboratory (LIO), which is affiliated with the ÉTS and CRCHUM.

**Figure 4:**
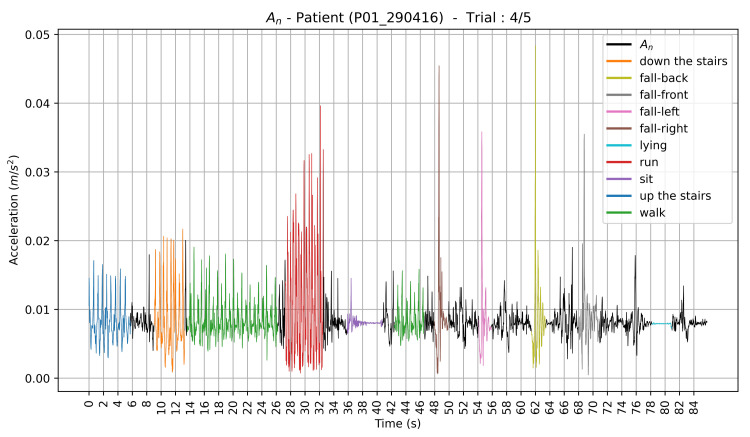
Plot of the raw normalized and labeled accelerometer data corresponding to one trial of a given participant (P01_290416). Each data cluster corresponding to an activity label has a different color on the plot.

Let $A_{x}$, $A_{y}$ and $A_{z}$ denote, respectively, the accelerometer data along the *x*-axis, *y*-axis and *z*-axis. We also computed the Euclidean norm $A_{n}$ of the three-axis acceleration signal (Figure 4):

(1) $$A_{n}=\sqrt{A_{x}^{2}+A_{y}^{2}+A_{z}^{2}}.$$

The data are represented in a csv file (the dataset *D*) in a structured format, with the following attributes

- Index: line number in the csv file (N=3.5 millions data points);
- Participant number, in {1,…,44};
- Participant reference: nomenclature as saved in the LIO server PXX_ddmmYY;
- Trial number, in {1,…,6};
- Timestamp YYYY−mm−ddHH:MM:SS.FFF (format in milliseconds);
- $A_{x}$: data from the *x*-axis accelerometer sensor;
- $A_{y}$: data from the *y*-axis accelerometer sensor;
- $A_{z}$: data from the *z*-axis accelerometer sensor;
- $A_{n}$: euclidean norm;
- activity name.

**Figure 5:**
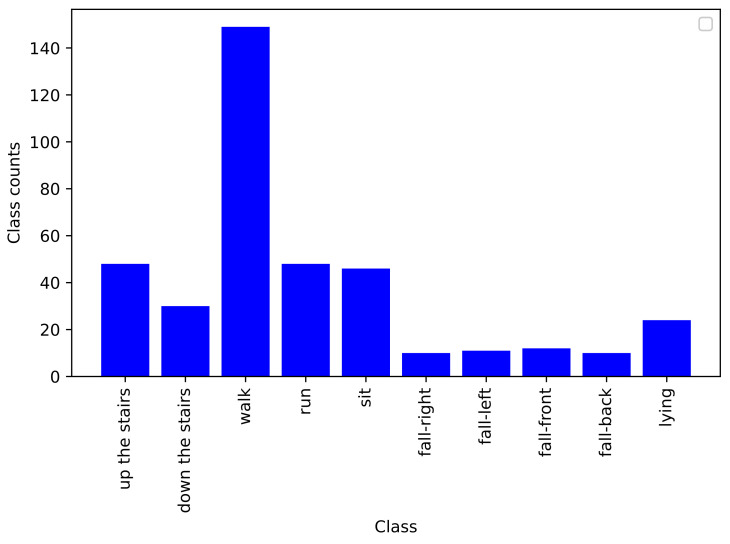
Bar chart of class distribution of all trials of a given participant (P01_290416), providing a strong visual indication of class imbalance in the dataset.

Since this dataset represents activities in real contexts, class imbalances occur. For example, as can be seen in Figure 5, there are more instances of walking than other activities.

#### 3.2.2. Validation

The validation was performed using leave-one-subject-out cross-validation of the dataset [40], using the scikit-learn package. The dataset was split according to the number of subjects in the dataset—that is, in each fold the model, was trained on all the subjects except one, which was used for testing. In this case, the number of folds is equal to the number of subjects in the dataset. In order to have an accurate estimation of the proposed method performance, this procedure was repeated until all the subjects had been used as test datasets. Obviously, activity patterns are subject-dependent—that is, the performed activities vary considerably among subjects according to their personal styles and anthropometry, which we refer to as inter-subject variability. Moreover, the way a given activity is performed by a given subject at different times may show variations, which we refer to as intra-subject variability. In our work, the intra-subject variability was considered when the participants were instructed to repeat the sequence of activities a given number of times (i.e., the number of trials). Using the leave-one-subject-out strategy guarantees that data from the same subject are either present in the train set or in the test set. This is a subject independent (inter-subject) validation technique to estimate generalization capabilities of learning techniques, and also allows performance analysis results per subject. We note that the number of samples slightly varies from one subject to another. For instance, for the subject having the reference P01_290416 and for whom we displayed the acceleration data in Figure 3 and Figure 4, there are 284,516 segments in the training set and 1552 in the testing set.

#### 3.2.3. Segmentation

Here, the input data ($A_{x}$, $A_{y}$, $A_{z}$, and $A_{n}$) are chronologically sequenced to form multivariate time series signals (of length *N*). We segmented these raw and continuous data flow signals using a 1-s fixed-size overlapping sliding window (FOSW), into *K* fixed-length segments, with a 50% overlap, in order to search for useful patterns in the time series. The window size T = 1 s corresponds to 64 data points and was chosen according to the accelerometer frequency (64 Hz). The segments were stored in a three-dimensional tensor of size K×T×m, where *K* is the number of segments, *T* is the time step (window size), and *m* is the number of multivariate time series. The three-dimensional tensor was used as the input of the classifier. The tensor-based approach allows one to reduce the computational cost when dealing with big data. Here, m=4, which corresponds to $A_{x}$, $A_{y}$, $A_{z}$ and $A_{n}$.

In temporal segmentation, the problem of impure segments may occur. Since for the transition points between activities, a segment is composed of more than one label [41]. To overcome the multiclass segment problem, ambiguous segments, that contain more than a label, were discarded. We also note that we have performed the train/test split before the segmentation, in order to avoid train and test data overlapping.

### 3.3. Overview of the Proposed Architecture

We considered a multiclass pattern classification task, where the input (i.e., multivariate time series signals) is to be classified into one, and only one, of the *l* non-overlapping classes referring to the considered human physical activities in Section 3.

**Figure 6:**
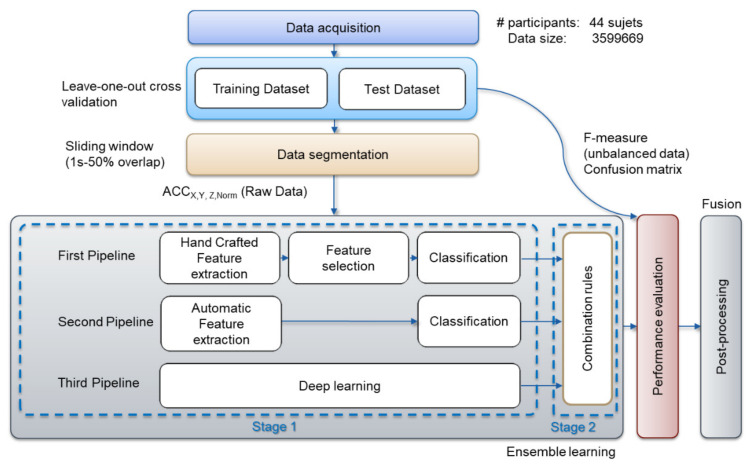
Overview of the proposed algorithm architecture.

The main contribution of this study is the development of a reference architecture design based on an ensemble learning system that uses both machine learning and deep learning techniques on a large dataset in order to achieve the highest generalization performance. Moreover, a parallel training approach for the proposed architecture was developed to accelerate the cross-validation procedure by running multiple independent training tasks on multiple cores or processors in parallel. In other words, we built an ensemble learning system with an efficient cross-validation implementation using parallel processing in order to balance the generalization performance and to reduce the computational cost of the system. Figure 6 shows the proposed ensemble learning architecture. The proposed ensemble learning architecture combines three data representation-based approaches. In the remainder of the paper, the process of tying together different learning algorithms is referred to as a pipeline. The pipeline ensures that the base classifiers to be combined later in an ensemble are diverse, i.e., independent of each other. The first pipeline encompasses feature engineering-based classifiers, in which features are manually extracted and later processed by machine learning algorithms. The second and third pipelines encompass feature learning-based classifiers. In the former, features are automatically extracted from the raw accelerometer data without relying on domain knowledge using a basic form of feature learning. In the latter, we explored deep learning-derived features from the raw accelerometer data. In each pipeline, we selected three classifiers that are commonly used for practical activity recognition. Moreover, this architecture is tractable so as to allow easily the inclusion of additional classification models inside each pipeline—that is, each pipeline in the proposed ensemble learning architecture could be extended by evaluating more than one standard learning algorithm belonging to each approach, on the data at hand, and fusing their predictions to achieve a better recognition rate, instead of only retaining the best classifier and discarding those providing a lower accuracy. We should note that the adopted learning algorithm used within each pipeline serves as a proof of concept of the proposed ensemble learning architecture. Ultimately, this approach not only allows us to compare heterogeneous classifiers, but also combines their predictions, thus decreasing the risk of selecting an inadequate single classifier—that is, a decision fusion method, as explained in Section 2, was used to yield the final prediction results [28] in order to combine the resulting three pipeline decisions.

Moreover, the proposed architecture deals with multivariate accelerometer time series data. Hence, in each stand-alone classifier, we separated multivariate time series into univariate ones ($A_{x}$, $A_{y}$, $A_{z}$ and $A_{n}$ separately, i.e., data fusion method), and we performedeither feature engineering or feature learning on each univariate time series individually. Axis-based feature fusion concatenatesdifferent resulting features from all the univariate time series inputs before the classification [25].

#### 3.3.1. First Pipeline

The first pipeline encompasses feature engineering-based classifiers, in which time and frequency domain features were manually extracted, then relevant ones were selected and later processed by base-level classifiers. In this case, we use ReliefF for feature selection combined with a Support Vector Machine (SVM).

The motivation for using time and frequency domain features in our architecture is that they are often used in practical activity recognition algorithms due to their simplicity to setup and their lower computational complexity [5]. Moreover, time domain features show how the signal changes in time. Frequency domain features show the distribution of the signal energy and are used to capture the repetitive nature of sensor signals [30]. We also note that the usefulness of the time and frequency domain features has been demonstrated in a prior study [39] on the convenience sample.

Furthermore, we decided to use ReliefF for feature selection due to its advantages in terms of both the learning time and the accuracy of the learned concept, as well as its practicality [42]. We used SVM, a well-known high performance classifier method, as a classifier in the first pipeline since the combination of the ReliefF-based feature selection and the SVM-based machine learning algorithm has been previously investigated by our research group [39] and gave promising results on the convenience sample.

##### Handcrafted Feature Extraction

For each feature univariate time series, commonly used time and frequency domain metrics were computed for each segment to extract basic signal information from raw accelerometer data [30].

- Time domain metrics: mean, variance, standard deviation, maximum, minimum, Root Mean Square (RMS), kurtosis, skewness, euclidean norm and l1-norm;
- Frequency domain metrics: energy and maximum magnitude of the Fast Fourier Transform (FFT).

Moreover, the cross-correlation was computed in a pairwise fashion between two features (i.e., ($A_{x},A_{y}$), ($A_{x},A_{z}$), ($A_{y},A_{z}$)) … on each segment.

The handcrafted feature extraction procedure leads to a total of 55 time-frequency features per segment that were z-score normalized.

##### Feature Selection Using ReliefF

The resulting features were ranked using the ReliefF feature selection algorithm [43]. The algorithm is based on computing the importance of the features by randomly choosing a given number of instances in the dataset (this number is a user-defined parameter) and searching for its two nearest neighbors: one from the same class and the other from a different class.

##### Multiclass Support Vector Machine (SVM) Classifier

The next step in this classification pipeline is the use of the SVM, which is a supervised machine learning algorithm [44]. For the binary classification SVM, the idea is to construct a hyperplane to separate the two classes so that the distance between the hyperplane and the sample points is maximal.

One approach to solve the *l*-class SVM (l>2) problem is to consider the problem as *l* binary SVMs. This approach is called a one-vs.-all SVM. Another approach consists of considering the problem as l×(l−1)/2 binary classifiers using all the binary pair-wise combinations of the *l* classes. This approach is called one-vs.-one SVM. In this study, we considered the one-vs.-all SVM. The kernel (i.e., the type of hyperplane used to separate the data), gamma (i.e., a parameter for non-linear hyperplanes), and C (i.e., the penalty parameter of the error term) are the hyperparameters of SVM, which need to be fine-tuned. In our case, we manually tuned these hyperparameters, we used the Radial Basis Function (RBF) kernel SVM and we set gamma to 0.001 and C to 1000.

#### 3.3.2. Second Pipeline

The second pipeline encompasses feature learning-based classifiers, in which features are automatically extracted from the raw accelerometer data without relying on domain knowledge using a basic form of feature learning and are later processed by base-level classifiers. We used Linear discriminant analysis (LDA) as an automatic feature extractor for the following advantages. It is a traditional statistical technique that reduces dimensionality while preserving as much of the class discriminatory information as possible [45]. Unlike Principal Component Analysis (PCA), which tries to maintain data information as much as possible, LDA is able to make the data points more distinguishable after dimension reduction [6]. We selected KNN as our base-learner classifier in this pipeline following Bicocchi et al. [33], who used the KNN algorithm to infer user activities on the basis of accelerometer data acquired from body-worn sensors and highlighted that it exhibits a state-of-the-art performance, is simple to implement, and can run on resource-constrained sensor platforms. Furthermore, they showed that it can achieve remarkable precision and accuracy levels in classifying simple and specific activities.

##### Automatic Feature Extraction Using LDA

Automatic feature extraction consists of mapping the raw data onto a lower dimensional space. LDA is a supervised feature extraction algorithm that takes the labels of the training data into account and aims at maximizing the class discrimination on the projected space [45]. The LDA algorithm was applied to each feature (i.e., the univariate time series). Then, the feature fusion concatenated the different transformed features to use them as an input for the *K*-nearest neighbor (K-NN) classifier.

##### K-NN Classifier

The principle of the K-NN algorithm is to find the closest *k* in distance training samples to the current sample and predict its label based on these *k* neighbors [46]. Generally, the value of *k* is specified by the user. The distance can, in general, be any metric measure. We note, however, that the standard Euclidean distance is the most common choice. The optimal choice of *k* is highly data-dependent and needs to be fine-tuned.

#### 3.3.3. Third Pipeline

We adopted a convolutional neural network (CNN) architecture for multivariate time series trained for both feature extraction and classification.

##### CNN for Multivariate Time Series

Let us first recall that a deep neural network has an input layer, an output layer and more than two hidden layers. A layer is a collection of neurons. A neuron takes a group of weighted inputs, applies a non-linear activation function, and returns an output.

- The input layer has T×m neurons.
- Hidden layers of a deep network are designed to learn the hierarchical feature representations of the data. During the training, a set of hyperparameters was manually tuned, and the weights were initialized randomly [47]. By gradient descent, the weights were updated using the back propagation algorithm in a way that minimizes the cost function on the training set. The choice of the model, the architecture and the cost function was crucial to obtain a network that generalizes well, and is in general problem- and data-dependent.
- The output layer has *l* neurons, which corresponds to the multiclass classification problem in this application.

In this work, we trained a CNN-based architecture. We recall that CNNs can capture the local connections of multimodal sensory data [48]. CNN combines three architectural ideas: local receptive fields, shared weights, and pooling, and it is based on two building blocks:

- The convolution block, which is composed of the convolution layer and the pooling layer. These two layers form the essential components of the feature extractor, which learns the features from the raw data automatically (feature learning).
  The convolutional layer implements the receptive field and shared weight concepts. Neurons in the convolutional layers are locally connected to neurons inside its receptive field in the previous layer. Neurons in a given layer are organized in planes where all the neurons share the same set of weights (also called filters or kernels). The set of outputs of the neurons in such a plane is called a feature map. The number of feature maps are the same as the number of filters. A pooling layer performs either an average subsampling (mean-pooling) or a maximum subsampling (max-pooling). For a time series, the pooling layers simply reduce the length, and thus the resolution, of the feature maps.
- The fully connected block, which performs the classification based on the learned features from the convolutional blocks.

The different hyperparameters of CNNs are the optimization algorithm (momentum), the number of epochs, the number of layers, the number of filters, the filter size, the activation function, the cost function, the batch size and the weight initialization [49].

**Figure 7:**
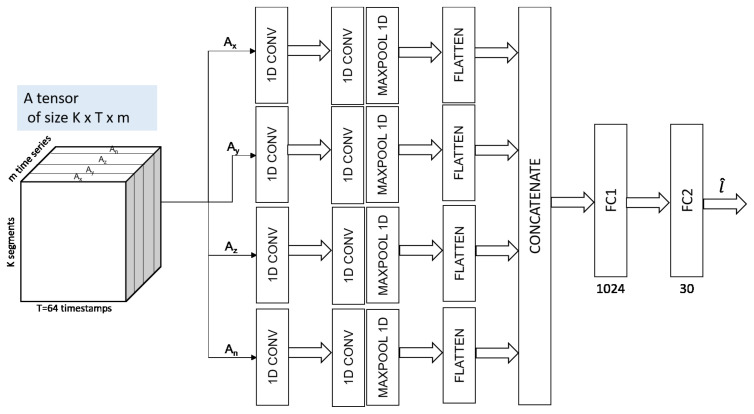
The three-dimensional tensor representation of the multivarite time series data is the input of the convolutional neural network pipeline of our architecture.

We applied a CNN model to the multivariate time series classification task at hand. First, we separated multivariate time series into univariate ones and performed feature learning on each univariate time series individually. Then, we concatenated the resulting features at the end of the feature learning step to conduct the classification. Our CNN-based model has three layers including two convolutional blocks, and two fully connected layers, as depicted in Figure 7—that is, for each feature, the input (i.e., univariate time series) is fed into a one-stage feature extractor (the convolutional block). The convolutional block consists of a convolution layer with three filters, a filter size of 20, a REctified Linear Units (ReLU) activation layer and a max pooling layer with pooling size of 3. At the end of the feature extraction step, we flattened the feature maps of each univariate time series and combined them as the input of subsequent fully connected block for classification. The fully connected block consists of two fully connected layers with 1024 and 30 neurons, respectively, with a ReLU activation function [23].

#### 3.3.4. Fusion Stage

Each pipeline computes a prediction vector for the same training dataset. The final decision is made by a combination of the three pipeline decisions (prediction vectors) using a decision rule (majority voting) [50] to produce the final classification result. We used majority voting for decision fusion because it is simple yet tractable, and can cope easily with a change in the number and type of classification models.

Here, we predict the final class label $\hat{l}$ based on the majority (plurality) voting of each classifier $l_{i}$:

$$\hat{l}=mode\left\{l_{1},l_{2},l_{3}\right\}$$

i.e., the final class label $\hat{l}$ corresponds to the class label that has been predicted most frequently by the three used classifiers within the three pipelines of our architecture.

#### 3.3.5. Computational Optimization

One of the issue to consider when designing and developing machine and deep learning applications is the computational cost. In fact, on the one hand, the more data we consider the more robust the application is, but, on the other hand, the more time consuming the application is as well, regarding both the data processing and the best model building and selection. To overcome such an issue, one should consider a more efficient usage of the computing resources by considering parallel and/or distributed programs. For example, the leave-one-subject-out cross-validation that we used in our architecture is computationally expensive. However, since the tasks performed on each fold are completely independent from the rest of the remaining folds, we ran as many folds as the core number of the target architecture in parallel. To do so, we used the multiprocessing parallel python package [51]. For the parallel implementation of the leave-one-subject-out cross-validation, we developed our own version. There are some other parallel versions of this method [52] but, to the best of our knowledge, such parallel versions have not been used in the context of HAR systems, probably because the training datasets were not as large as our dataset. We also note that in order to further enhance the performance of our system, we used a GPU accelerator to run the mutivariate CNN model from the python keras package with tensorflow backend [53]. To ensure a good balance between the generalization performance of the model and its computational performance, the different hyperparameters of the deep learning model such as the batch_size should be carefully chosen [54]. In this application, we first used the python multiprocessing package which allows, just by adding a few lines of code, to run up to 5× faster comparing to the sequential code using a machine with 8 cores and 1 NVIDIA TITAN RTX GPU. A more detailed description of the machine will be given in the following section.

## 4. Results

### 4.1. Experimental Design

#### 4.1.1. Weighting Imbalanced Classes

To handle the class imbalance problem, we do not include any oversampling but an algorithm-level method—that is, class weight is added that automatically assigns higher weights to the minority classes in the learning process, in order to reduce bias towards the majority group [55].

#### 4.1.2. Multiclass Performance Measures

The result of an *l*-class classification can be visualized in a confusion matrix of size l×l. Each row in a confusion matrix represents an actual class, while each column represents a predicted class. By definition, each entry $C_{ij}$ in a confusion matrix C denotes the number of observations (segments) from class *i* predicted to be of class *j*.

The recall, precision and F1-score are used as performance metrics to evaluate the correctness of a classification. The F1-score metric is particularly interesting for its robustness to class imbalance [56]. The recall ($R_{i}$), precision ($P_{i}$) and F1-measure ($F1_{i}$) for class *i* in a multiclass problem can be defined by the following equations,

(2) $$P_{i}=\frac{TP_{i}}{TP_{i}+FP_{i}},\;\;R_{i}=\frac{TP_{i}}{TP_{i}+FN_{i}},\;\;\mathrm{and}\;\;\;F1_{i}=2\times \frac{P_{i}\times R_{i}}{P_{i}+R_{i}},$$

where $TP_{i}$ is the number of objects from class *i* assigned correctly to class *i*, $FP_{i}$ is the number of objects that do not belong to class *i* but are assigned to class *i*, and $FN_{i}$ is the number of objects from class *i* predicted to another class.

The quality of the overall classification is usually assessed in two ways: macro-averaging and micro-averaging. The first computes the measure separately for each class and then takes their unweighted mean. A weighted average could be computed by support (i.e., the number of true instances for each label) to account for class imbalance. The second calculates the measure globally by counting the total true positives, false negatives and false positives.

In the following equations, κ and μ indices refer to the macro- and micro-averaging, respectively. *P*, *R*, and F1 are the total precision, recall and *F*1 measures [56].

(3) $$P_{\kappa }=\frac{1}{K}\sum_{i=1}^{K}P_{i},\;\;R_{\kappa }=\frac{1}{K}\sum_{i=1}^{K}R_{i},\;\;\mathrm{and}\;\;\;F1_{\kappa }=2\times \frac{P_{\kappa }\times R_{\kappa }}{P_{\kappa }+R_{\kappa }}.$$

(4) $$P_{\mu }=\frac{\sum_{i=1}^{K}TP_{i}}{\sum_{i=1}^{K}\left(TP_{i}+FP_{i}\right)},\;\;R_{\mu }=\frac{\sum_{i=1}^{K}TP_{i}}{\sum_{i=1}^{K}\left(TP_{i}+FN_{i}\right)}\;\;\mathrm{and}\;\;\;F1_{\mu }=2\times \frac{P_{\mu }\times R_{\mu }}{P_{\mu }+R_{\mu }}.$$

### 4.2. Experimental Results

The classification was performed using a leave-one-subject-out cross-validation as detailed in Section 3.2.2. Instead of averaging the performance measures of each holdout fold, predictions were computed and stored in a list. Then, at the end of the run, the predictions were compared to the expected values for each holdout test set and a single performance measure is reported.

#### 4.2.1. Classification Result Using the Handcrafted Feature Engineering Based Approach

**Figure 8:**
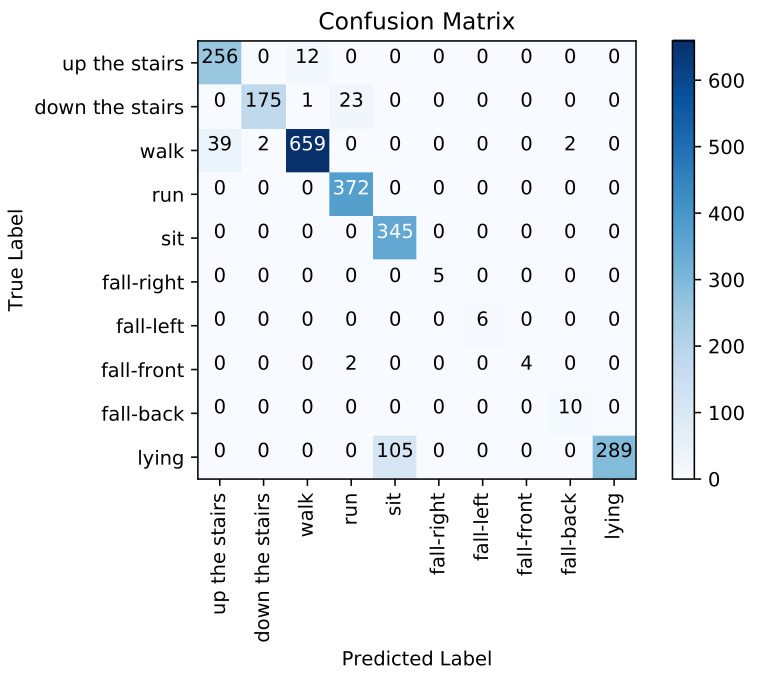
Confusion matrix using the handcrafted feature engineering based approach.

Figure 8 presents the confusion matrix for inter-subject activity recognition obtained using the adopted method with time and frequency domain features, ReliefF feature selection, and SVM classifier (first pipeline). Table 1 shows medium to high precision scores (from 77% to 100%), medium to high recall scores (from 67% to 100%), and high F1-scores (from 80% to 100%) on each activity class. The overall weighted averaged precision, recall, and F1-score across all activities are 93%, 92%, and 92%, respectively.

If we examine the recognition performance for each activity individually, walking up the stairs is confused with walking (0.04%), walking down the stairs is confused with running (0.11%), walking is confused with walking up the stairs (0.26%), and lying is confused with sitting (0.27%).

#### 4.2.2. Classification Result Using the Automatic Feature Extraction-Based Approach

**Figure 9:**
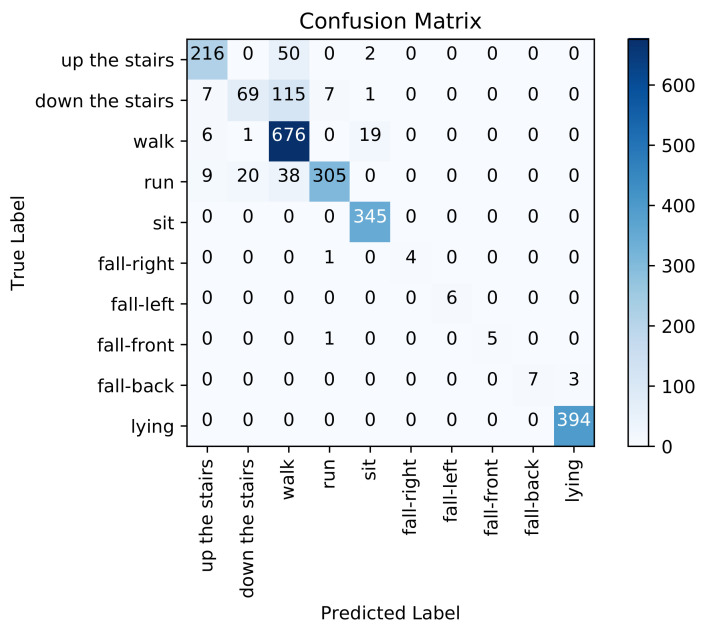
Confusion matrix using the automatic feature extraction based approach.

Figure 9 presents the confusion matrix for inter-subject activity recognition obtained using the adopted automatic feature extraction based on LDA and KNN classifier (second pipeline). Table 2 shows medium to high precision scores (from 77% to 100%), low to high recall scores (from 35% to 100%), and low to high F1-scores (from 48% to 100%) on each activity class.

The overall weighted averaged precision, recall and F1-score, across all activities, are 88%, 88%, and 87%, respectively. If we examine the recognition performance for each activity individually, we can see that, again, walking up the stairs is confused with walking (0.18%), walking down the stairs is confused with walking (0.77%), walking is confused with sitting (0.02%), and running is confused with walking up the stairs (0.02%), down the stairs (0.05%) and walking (0.10%). We notice that this approach performs worse than the previous approach on dynamic activities (walking, walking up and down the stairs and running). However, it performs better on static activities (sitting and lying).

#### 4.2.3. Classification Result Using Feature Learning-Based Approach

**Figure 10:**
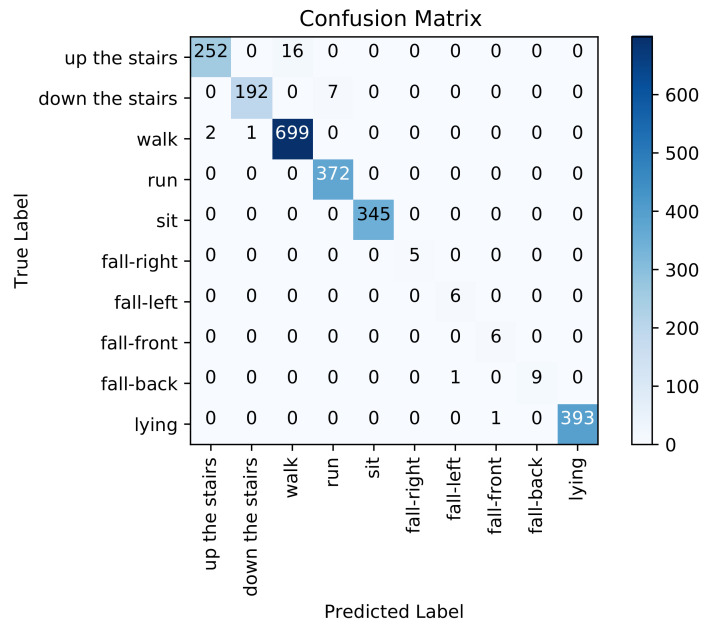
Confusion matrix using feature learning based approach.

Figure 10 presents the confusion matrix for inter-subject activity recognition obtained using the adopted multivariate CNN classifier (third pipeline). Table 3 shows high precision (from 86% to 100%), high recall (from 90% to 100%), and high F1-score (from 92% to 100%) on each activity class. The overall weighted averaged precision, recall, and F1-score across all activities are all equal to 99%. If we examine the recognition performance for each activity individually, walking up the stairs is confused with walking (0.05%), walking down the stairs is confused with running (0.03%), and walking is confused with walking up the stairs (0.002%) and down the stairs (0.001%). We notice that this approach performs better than the two previous approaches on recognizing both dynamic and static activities.

#### 4.2.4. Classification Results Using the Ensemble Learning Based Approach

**Figure 11:**
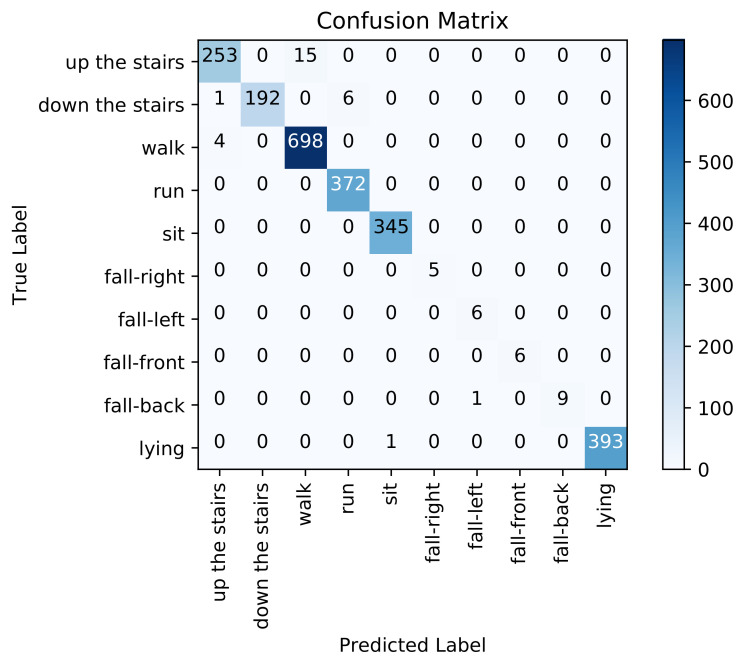
Confusion matrix using the ensemble learning-based approach.

Figure 11 presents the confusion matrix for inter-subject activity recognition obtained using the proposed ensemble learning approach, which combines the results of the three previous pipelines. Table 4 shows high precision (from 86% to 100%), high recall (from 90% to 100%) and high F1-score (from 92% to 100%) on each activity class. The overall weighted averaged precision, recall and F1-score across all activities are about 99%. If we examine the recognition performance for each activity individually, walking up the stairs is confused with walking (0.05%), walking down the stairs is confused with running (0.03%), and walking is confused with walking up the stairs (0.002%). The proposed ensemble learning approach performed extremely well in recognizing the ten different activities compared to the three previous approaches considered individually.

### 4.3. Discussion of the Recognition Rate Results

In this paper, we focus on the recognition of three types of human physical activities (static activity, dynamic activity, and hazardous situations such as falling). Each of the three tested approaches has its own strengths and weaknesses when classifying different types of activities. Sitting and lying are static activities. Dynamic activities include walking, walking up and down the stairs and running. Concerning dynamic activities, we recall that walking generates a periodic pattern and running implies a motion similar to walking, but is executed faster. Looking at Figure 3, a periodic pattern can also be seen for running, with a shorter time difference between periods compared to walking. Some false positives and negatives showed up when trying to distinguish and recognize these two activities using the automatic feature extraction-based approach. Moreover, walking and walking up and down the stairs are almost similar (see Figure 3) and can be performed in many ways. Thus, they are easily confused. However, the proposed ensemble learning model performed extremely well when identifying the different dynamic activities compared to standalone approaches.

Concerning static activities, We note that lying down and sitting are confused in the handcrafted feature engineering approach since the orientation of the waist-worn accelerometer is similar for both activities. We also note that the proposed models were trained with less lying and sitting data than for others classes (as depicted in Figure 5), which makes it even more difficult for the models to learn them. As described in the confusion matrices in Figure 8, Figure 9, Figure 10 and Figure 11, we can see that for the four fall classes, labeled observations are scarce compared to the other classes such as walking and running (see Figure 5). This is explained by the short duration of the falls. The consideration of this class in our system aims at detecting a fall if it occurs during the rehabilitation process. We recall, however, as previously explained in the protocol, that the falls we are considering here are simulated and may be different from an actual fall. We also consider four classes of falling to show that our system is able to capture the direction of the fall, which is very important since falling front or back have in general a more important impact.

**Figure 12:**
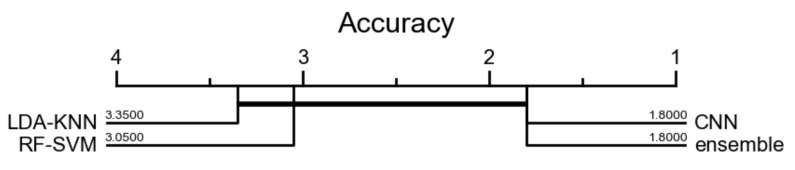
Statistical comparison of the 4 models over the physical activities classes. Average ranks of examined models are presented. A thick horizontal line shows a group of classifiers that are not significantly different in terms of accuracy.

In order to compare the four models over the physical activities classes, following Fawaz et al. [57], we conducted statistical analysis by using the Friedman test to reject the null hypothesis. The test addresses the hypothesis that all methods perform equally well. For the post hoc analysis, following the recent recommendations in [58], we perform the pairwise comparisons of the post hoc analysis using the Wilcoxon signed rank test instead of the average rank comparison. In order to visualize the comparison, we used a critical difference diagram [59] to display the results of these statistical tests projected onto the average rank axis, with a thick horizontal line showing a of classifiers that are not significantly different [57]. Following Fawaz et al. [57], we perform the pairwise comparisons of the post hoc analysis using the Wilcoxon signed rank test [58]. We used the Python code provided in [57]. The corresponding critical difference diagram is depicted in the Figure 12 below, where the statistical test failed to find any significant difference between all 4 models. Indeed, the CNN classifier results are very close to those obtained using the whole ensemble learning architecture. We also noticed that the two other approaches correct misclassification errors of the feature leaning approach with CNN. Moreover, the proposed architecture allows us to understand the learned features by each proposed approach (feature engineering and feature learning) and compare the corresponding output feature vectors, to counterbalance interpretability and generalization.

### 4.4. Performance Speed Analysis

Here, we present the computational cost of the parallel HAR system we developed. We ran our experiments on a machine with an intel Core *i*7-9700 processor having 8 cores and a 64 GB RAM connected to a GPU accelerator of type NVIDIA TITAN RTX. One of the bottlenecks in terms of running time in our system corresponds to the segmentation stage. In fact, running a sequential segmentation (using only one core of the cpu) relative to the 44 participants based on the leave-one-subject-out validation technique takes around 6.5 days. However, when we ran the segmentation in parallel making full usage of the 8 cores of our machine via a parallel implementation based on the multiprocessing python package, it takes around 32 h, which is almost 5× faster than the sequential version. As described in Section 3.3.5, each fold training relative to one of the train/test split was run as an independent task. Thus, a task queue formed and the different tasks were assigned to the 8 cores of the machine. Each task is a succession of the three pipelines of our learning models applied to the considered fold. Here again, we used the multiprocessing python package, and we obtained a speed up of almost 3 compared to the sequential implementation. We also note that, within each task, the third pipeline, which corresponds to the multivariate CNN method, was run on the GPU accelerator, which takes around 15 s, which is 80× faster than a purely cpu version, which takes around 20 min. We mention here that given that each task is running on an independent data subset, our performance results regarding the computational time could be further improved by using a larger number of cores and/or processors.

### 4.5. Comparison Results

To validate the collected dataset using the Hexoskin t-shirt, we considered the previously available WISDM dataset as a benchmark dataset and compared its results to those of the collected dataset. The WISDM dataset is used for human activity recognition to classify different physical activities using cell phone accelerometers [12]. It contains accelerometer data collected from 36 subjects performing six activities, namely walking, jogging, ascending stairs, descending stairs, sitting, and standing. These subjects carried an Android phone in the front pocket of their pants leg when performing these activities. We recall that the collected hexoskin dataset is 2.5× larger than the WISDM dataset. We also compare the ensemble approach that we proposed to a selection of classifier algorithms including an ensemble approach, referred to as ensemble (DT-LR-MLP), that combines J48 decision tree, Multilayer Perceptrons (MLPs) and Logistic Regression [38] along with Random Forest [60] and Adaboost [61] using the default parameters available in the sklearn library. To ensure a fair comparison, we applied the selected classifiers to both the WISDM and the Hexoskin datasets following the same process as in the proposed approach described throughout this work by:

- Partitioning the dataset into training and testing subsets using the leave-one-subject-out cross-validation;
- Segmenting the raw accelerometer signals, using a 1-s fixed-size overlapping sliding window (FOSW), with a 50% overlap;
- Handling class imbalance in all the learning techniques;
- Making a comparative analysis on the basis of performance measures such as F1-score, precision, and recall as well as confusion matrices using the mean score estimated on each group of out-of-fold predictions.

Table 5 represents the classification performance for different classifiers on both the collected Hexoskin dataset and the WISDM dataset. The obtained results show that the proposed ensemble learning-based heterogeneous machine learning and deep learning algorithms applied to the collected dataset using the Hexoskin t-shirt outperforms the other classifier algorithms used. These results are displayed in bold in Table 5.

## 5. Discussion

The main goal of this research work is the development of an adherence measurement system that is completely objective, precise and efficient in terms of computational resource usage. This requires the development of a recognition system for human physical activity, which is what we focus on in this paper. In the following, we summarize our contributions and the limitations of the proposed method. Then, we present some possible perspectives and insights.

The first part of this research is the acquisition of a large multimodal dataset based on a wearable sensor vest that captures cardiac, respiratory, and accelerometer data from healthy and young volunteers. Each participant undergoes a sequence of ten real-life physical activities, including static and dynamic activities, that are likely to occur during a patient rehabilitation protocol [62]. The purpose of this data acquisition is to establish a proof of concept that the recorded acceleration data from the waist-worn accelerometer during physical activity could be modeled for a HAR system using learning techniques for the purpose of health monitoring in an upcoming stage of a more global research project.

The second part is the development of a recognition system for these human physical activities using accelerometer data collected from a waist-mounted accelerometer in the vest. We note that the model may be extended by some other activities. Later, the pretrained HAR system should be able to classify activities of patients undergoing a cardiac rehabilitation program. The developed HAR system is based on an ensemble learning architecture that combines different data representation-based classifiers (feature engineering and feature learning). The output of these classifiers are combined to improve the classification performance (minimizing false positives and false negatives). An inter-subject validation strategy (leave-one-subject-out cross-validation) is used to make a realistic estimate of the performance generalization of each classifier independently, as well as the ensemble architecture. However, classifier ensembles combined with the leave-one-subject-out validation technique are clearly more expensive on large datasets, computationally speaking, as they require several models to be trained. Hence, we propose a parallel implementation for our architecture to accelerate the cross-validation procedure by running multiple fold trainings simultaneously. We also enhance the computation relative to each fold by the use of a GPU accelerator. Another advantage of our implementation is the fact that the segmented multivariate time series are stored into a fourth-order 3D tensor. Finally, we demonstrate that forms of locomotion such as walking, running, and climbing the stairs, and postures such as sitting and lying down, as well as some hazardous situations such as falling, can be recognized at up to a 99% recognition rate using the waist-worn accelerator.

Nevertheless, this study has some limitations. For example, in this work, we focus on classifying activities collected in a laboratory environment. Thus, the population sample includes only young and healthy subjects. We should train our system on a heterogeneous population sample (able-bodied, elderly, cardiovascular diseased patients) that can benefit from HAR. In fact, for the more general research project, it is important to train and test activity recognition systems on data collected under naturalistic circumstances, with patients undergoing a rehabilitation program. We note that we planned to collect data from a pathological population following a cardiac rehabilitation program in collaboration with the Centre de cardiologie préventive (CCP) in the Centre Hospitalier de l’Université de Montréal (CHUM) to rigorously validate the efficiency of the proposed HAR system. Unfortunately, the recruitment process has been interrupted by the pandemic circumstances.

In addition, ground truth data have been semi-automatically annotated. The annotation procedure may be fine-tuned, and a real-time automatic annotation may be investigated in order to increase the volume of data collected. Moreover, we do not take into account transitional activities neither in the segmentation nor in the classification processes. Transition-aware and activity-based segmentation approaches could be investigated.

Here, we examine the similarities and differences between this work and other ensemble learning methods for HAR [37,63]. The reviewed methods use different datasets. Therefore, the reported accuracies and execution times cannot be compared. For instance, Rahim et al. [63] discussed the performance of five types of ensemble classifiers: bagging, adboost, rotation forest, ensemble of nested dichotomies, and random subspace, all with either support vector machine (SVM) or random forest (RF) as the basic learning scheme. Two open datasets were considered, containing, respectively, 5447 and 10,299 samples, collected, respectively, on 31 and 30 participants using smartphone inertial sensors. They also used 17 features from the time and frequency domains. Data classification is evaluated with the holdout (70–30%) and 10-fold cross-validation methods. They observed that, overall, SVM produced better accuracy rates, reaching 99.22% compared to RF with 97.91%. The authors do not discuss computational cost or implementation aspects. Rahim et al. mainly focused on data sampling methods, where they considered the five different strategies mentioned above. In this study, we use *K*-fold cross-validation, with *K* being the number of subjects in the dataset, that is 44, as the resampling method. To make a fair comparison, we intend to test other resampling methods, such as random splits and bootstrap aggregation, more commonly referred to as bagging. As to training, Rahim et al. used solely handcrafted feature-based classifiers (SVM and RF), while we consider both feature engineering and feature learning approaches. Moreover, our dataset is significantly larger (3.5 millions samples) than the two datasets they use for the performance evaluation. Similar to our study, Xu et al. [37] applied cascade ensemble learning (called CELearning) to model human activity recognition. However, they applied feature fusion of handcrafted and automatically extracted Fast Fourier Transform (FFT) features before classification.

In the following, we enumerate seven possible directions for future work. The first is to assess performance measures separately for different demographic categories and collect more training data while ensuring diversity of the demographic characteristics, in order to ensure the diversity of our data to mitigate any bias. The second is to automatically tune the hyperparameters of the used learning algorithms in this paper in order to ensure the best performance when using our dataset. The third is to investigate open-source feature engineering libraries (for instance, tsfel [64] and tsfresh [65]) for time series, to capture as many discriminative signal characteristics of human physical activities as possible. The fourth is to understand the learned features by each proposed approach (feature engineering, automatic feature extraction and feature learning) by applying a Class Activation Map (CAM) [66], for example, and then comparing the corresponding output feature vectors. The fifth is to smooth and fuse the predicted activity labels of consecutive segments. Thus, identifying the start and end points of each activity is very useful for physical rehabilitation, to have better information about activity transitions and the duration of each activity. Moreover, each pipeline in the proposed ensemble learning architecture could be extended by evaluating more than one standard learning algorithm belonging to each approach, on the data at hand, and fusing their predictions to achieve a better recognition rate. We should note that the adopted learning algorithms used at each pipeline serve as a proof of concept of the proposed ensemble learning architecture. Finally, we intend to compare different classifier fusion methods such as bagging and boosting.

## Figures and Tables

**Table 1:** Classification report using the handcrafted feature engineering-based approach.

Class	Precision	Recall	F1-Score	Support
up the stairs	0.87	0.96	0.91	268
down the stairs	0.99	0.88	0.93	199
walk	0.98	0.94	0.96	702
run	0.94	1.00	0.97	372
sit	0.77	1.00	0.87	345
fall-right	1.00	1.00	1.00	5
fall-left	1.00	1.00	1.00	6
fall-front	1.00	0.67	0.80	6
fall-back	0.83	1.00	0.91	10
lying	1.00	0.73	0.85	394
macro avg	0.94	0.92	0.92	2307
weighted avg	0.93	0.92	0.92	2307

**Table 2:** Classification report using the automatic feature extraction based approach.

Class	Precision	Recall	F1-Score	Support
up the stairs	0.91	0.81	0.85	268
down the stairs	0.77	0.35	0.48	199
walk	0.77	0.96	0.86	702
run	0.97	0.82	0.89	372
sit	0.94	1.00	0.97	345
fall-right	1.00	0.80	0.89	5
fall-left	1.00	1.00	1.00	6
fall-front	1.00	0.83	0.91	6
fall-back	1.00	0.70	0.82	10
lying	0.99	1.00	1.00	394
macro avg	0.93	0.83	0.87	2307
weighted avg	0.88	0.88	0.87	2307

**Table 3:** Classification report using feature learning-based approach.

Class	Precision	Recall	F1-Score	Support
up the stairs	0.99	0.94	0.97	268
down the stairs	0.99	0.96	0.98	199
walk	0.98	1.00	0.99	702
run	0.98	1.00	0.99	372
sit	1.00	1.00	1.00	345
fall-right	1.00	1.00	1.00	5
fall-left	0.86	1.00	0.92	6
fall-front	0.86	1.00	0.92	6
fall-back	1.00	0.90	0.95	10
lying	1.00	1.00	1.00	394
macro avg	0.97	0.98	0.97	2307
weighted avg	0.99	0.99	0.99	2307

**Table 4:** Classification report using the ensemble learning-based approach.

Class	Precision	Recall	F1-Score	Support
up the stairs	0.98	0.94	0.96	268
down the stairs	1.00	0.96	0.98	199
walk	0.98	0.99	0.99	702
run	0.98	1.00	0.99	372
sit	1.00	1.00	1.00	345
fall-right	1.00	1.00	1.00	5
fall-left	0.86	1.00	0.92	6
fall-front	1.00	1.00	1.00	6
fall-back	1.00	0.90	0.95	10
lying	1.00	1.00	1.00	394
macro avg	0.98	0.98	0.98	2307
weighted avg	0.99	0.99	0.99	2307

**Table 5:** Comparison with state-of-the-art ensemble learning techniques applied to the WISDM and Hexoskin datasets.

Dataset	Methods	F1-Score	Precison	Recall
WISDM	The proposed method	**0.77 ± 0.07**	**0.77 ± 0.07**	**0.77 ± 0.07**
	Ensemble (DT-LR-MLP) [38]	0.73 ± 0.11	0.73 ± 0.11	0.73 ± 0.11
	Adaboost [61]	0.46 ± 0.13	0.46 ± 0.13	0.46 ± 0.13
	Random Forest [60]	0.72 ± 0.11	0.72 ± 0.11	0.72 ± 0.11
Hexoskin	The proposed method	**0.85 ± 0.12**	**0.85 ± 0.12**	**0.85 ± 0.12**
	Ensemble (DT-LR-MLP) [38]	0.79± 0.14	0.79± 0.14	0.79± 0.14
	Adaboost [61]	0.49 ± 0.11	0.49 ± 0.11	0.49 ± 0.11
	Random Forest [60]	0.81 ± 0.14	0.81 ± 0.14	0.81 ± 0.14

## Data Availability

The data presented in this study are available on request from the corresponding author. The data are not publicly available due to ethical and legal restrictions on consent given by research participants.
